# Nutritional Profile and Chlorophyll Intake of Collard Green as a Convenience Food

**DOI:** 10.3390/nu16234015

**Published:** 2024-11-23

**Authors:** Elisa Canazza, Paolo Tessari, Christine Mayr Marangon, Anna Lante

**Affiliations:** 1 Dipartimento di Agronomia, Alimenti, Risorse Naturali, Animali e Ambiente—DAFNAE, Università di Padova, Viale dell’Università, 16, 35020 Legnaro, PD, Italy; elisa.canazza@unipd.it (E.C.); christine.marangon@unipd.it (C.M.M.); 2 Senior Associate, University of Padova, Via Giustiniani 2, 35128 Padova, PD, Italy; paolo.tessari@unipd.it

**Keywords:** collard green, Couve-Manteiga, *Brassicaceae*, nutritional profile, chlorophyll, convenience food

## Abstract

**Background/Objectives:** Collard green (*Brassica oleracea* var. *viridis*) is widely cultivated for its adaptability and nutritional benefits. This study examines the nutritional composition and chlorophyll content of the “Couve-Manteiga” cultivar grown in Italy, emphasizing its potential application in convenience foods, such as fresh-cut, fifth-range, and freeze-dried products, to enhance chlorophyll intake in the population. **Methods:** The leaves of collard greens were analyzed for proximate composition, mineral content, amino acid and fatty acid profiles, and chlorophyll levels. Chlorophyll retention was measured after sous vide cooking and freeze-drying to assess the efficacy of these preservation methods. The chlorophyll content of different product formats was quantified, and potential dietary contributions were estimated based on consumption data. **Results:** Collard greens exhibited a low caloric value (30.66 kcal/100 g), with high levels of dietary fiber (3.39 g/100 g), protein (3.01 g/100 g), calcium (333.09 mg/100 g), and potassium (215.53 mg/100 g). The amino acid profile revealed an essential to non-essential amino acid ratio of 0.72. Chlorophyll retention was notably high in both freeze-dried (97.66%) and sous-vide cooked products (83.5%), indicating the effectiveness of these methods in preserving chlorophyll content compared to fresh-cut leaves. **Conclusions:** The results suggest that convenience foods made from collard green leaves provide an accessible means to boost chlorophyll intake and enhance daily nutrition, offering a practical solution for increasing the consumption of this nutrient-rich vegetable.

## 1. Introduction

*Brassicaceae*, commonly called Cruciferous, is one of the largest families of Angiosperms, with more than 360 genera and nearly 4000 species spread across several continents [1,2]. In recent years, Cruciferous vegetables have gained increasing attention for being an exceptional source of nutrients, such as proteins, vitamins, and minerals. The characteristic that distinguishes Cruciferous vegetables is the high content of glucosinolates, which convert into bioactive compounds with anticancer properties, such as isothiocyanates and indoles. Isothiocyanates actively contribute to protection against cancer by promoting the activation of phase 2 enzymes, involved in the detoxification of carcinogens, arresting cell cycle progression and inducing apoptosis of damaged cells. Studies in animal models have confirmed that isothiocyanates exert antiproliferative and pro-apoptotic effects through the accumulation of reactive oxygen species, crucial for the control of tumor growth [3]. Additionally, cruciferous vegetables stand out for their potent antioxidant effects, reducing oxidative stress and inflammation. Their diverse range of phytonutrients, including phenols and flavonoids, not only supports immune function but also provides antimicrobial and cardioprotective benefits. Moreover, their ability to influence detoxification pathways and promote cellular health adds to their exceptional profile [3,4,5].

Collard green (*Brassica oleraceae* var. *viridis*) is part of the Acephala group, which contains different morphotypes [6]. The name “Acephala” refers to a group of leafy cabbages “without head” [7]. The vegetables of the Acephala group of *Brassica oleracea* are biennial cultivars and originated in the Mediterranean region but have gained popularity worldwide due to their strong tolerance toward unfavorable environmental conditions, as well as their nutritional aspects and versatility in recipes [8]. Collard greens are known variously as *couve* (Brazil), *couve-galega* (Portugal), *kovi* or *kobi* (Spanish-speaking countries), *haak* (Kashmir), and *sukuma wiki* (East Africa). Popular cultivars of green collard include “Georgia Southern”, “Morris Heading”, and “Couve-Manteiga” [9,10].

The green color of plants is attributed to chlorophylls, the most abundant pigments on Earth, synthesized by plants, algae, and some bacteria. More than a hundred different structures of chlorophyll have been identified. The structures of chlorophyll a (Chl_a_) and b (Chl_b_) are the most abundant in green foods [11].

Interest in chlorophylls is increasing sharply, as recent studies have highlighted their ability to be absorbed and metabolized by the body and their protective role against carcinogens. The main mechanisms by which chlorophylls exert an antitumor action include antioxidant activity, which reduces oxidative stress and DNA damage, and the ability to complex mutagens in the gastrointestinal tract, limiting their systemic absorption and decreasing the risk of carcinogenesis. In addition, chlorophylls modulate the detoxification enzymatic pathways of xenobiotic compounds and can induce apoptosis in cancer cells, thereby contributing to their elimination and control of cancerous proliferation [12,13]. These aspects are increasingly attracting the attention of scientific research and the food industry [11,14,15,16].

Chlorophylls can undergo significant changes during the digestive process due to changes in pH and enzymatic reactions. This can lead to the pheophytinization and oxidation of ingested chlorophylls, transforming them into derivatives such as pheophytins and pheophorbides [14,17]. Despite these transformations, it is crucial to include sources of chlorophyll in the diet, as chlorophyll derivatives, once absorbed, can also benefit health. The ability of chlorophylls and their derivatives to be micellarized and absorbed changes, but even minimal absorption is physiologically significant, enhancing their protective potential in the body. Once micellized, these compounds can enter cells, where they exert protective effects through antioxidant activity, modulation of detoxification processes, and regulation of oxidative stress and inflammatory pathways [13,14].

Chlorophyll-derived color additives, identified as E140 and E141 [11], are authorized by European legislation and commonly used to provide stable and vibrant colors [18,19]. However, the food market is progressively evolving toward solutions that respect the concept of “clean label”, favoring “natural ingredients” that not only have coloring properties but also maintain the main compounds of the original plant-based food matrix. In response to these needs, research is focusing on plant sources rich in natural chlorophylls to develop concentrates in the form of juices, pastes, powders, and the like, which can be used as food coloring, replacing traditional natural or synthetic coloring additives [20,21]. Additionally, if these concentrates are obtained from plant by-products of the agrifood industry, it helps to reduce food waste and promote the circular economy of the food chain [22].

Among the drying methods, freeze drying is widely preferred for its ability to preserve food quality best. Studies have shown that freeze-dried leaves, such as kale and chives, maintain a significantly higher concentration of chlorophyll than other drying methods, with retention rates of up to 100% [23,24,25]. The use of these concentrated functional ingredients of chlorophylls and other bioactive components represents a promising way to increase the consumption of chlorophyll among the population, especially among the youngest, by proposing it in different and more palatable forms. Saidi et al. (2023) [26] have developed an artisanal ice cream with improved nutritional value thanks to the inclusion of green mustard leaves, both in powder and puree form. Waseem et al. (2024) [27] have obtained an unleavened bread enriched with spinach powder, significantly improving its nutritional and functional characteristics. Fanesi et al. (2023) [28] developed functional biscuits containing flour derived from broccoli by-products rich in vitamins, glucosinolates, carotenoids and chlorophylls.

A recent study on chronic intake of green chlorophylls in Europe, based on EFSA data, reported an average intake of 207.12 mg/day in European adults, with wide variations between countries and age groups. For example, in adults, the intake ranges from 44.40 mg/day in Denmark to 434.99 mg/day in the Netherlands. Similar ranges were observed for adolescents and the elderly. In the infant group, values range from 2.37 mg/day in Italy to 124.77 mg/day in France, with increased intake during adolescence. In Italy, the average consumption of chlorophyll increases with age, from 2.37 mg/day in children to 156.04 mg/day in adolescents, 152.28 mg/day in adults, and reaching 162.32 mg/day in the elderly [16]. In Italy, childhood obesity is among the highest in Europe [29], with 19% of children overweight and 9.8% obese. In addition, 25.9% consume fruit and vegetables less than once a day [30], far from the five portions recommended by the Italian guidelines and the World Health Organization (WHO) [31,32]. Even among adults, only 7% of Italians between 18 and 69 years of age include the recommended five portions of fruit and vegetables in their daily diet. In total, 52% of the population consumes only 1–2 servings a day, 38% takes 3–4 servings, and 3% consumes none [33]. Among the main obstacles that separate the modern consumer from an adequate consumption of vegetables are the cost, the lack of time for preparation, availability when eating out, preference, and social support [34,35,36]. However, the barriers related to preparation and cooking seem to have a greater impact than those related to purchase [34]. Plant-based convenience foods, with their high nutritional quality, offer a promising solution to improve diets and overcome the barriers related to vegetable preparation [36,37,38,39].

Several authors have documented the impact of domestic and industrial processing on the nutritional quality of *Brassicaceae*. Sous vide cooking has emerged as a method that offers numerous advantages over traditional techniques. It preserves vitamins and minerals, reduces oxidation, prevents moisture loss, and maintains volatile aroma [40,41,42,43,44]. Additionally, vacuum sealing inhibits bacterial growth, thereby prolonging the shelf-life of food products and reducing food waste. This is a crucial step in improving food safety in global food distribution and storage [38,44,45,46,47].

Starting from these considerations, this work aims to provide an in-depth analysis of the nutritional profile of the “Couve-Manteiga” cultivar grown in Italy.

Given its recent introduction into Italian agriculture, there is a notable absence of studies investigating the mineral, amino acid, and fatty acid profiles of this cultivar at a national level. This study addresses this gap by analyzing these aspects in depth, with a focus on the beneficial roles that these components play for consumers.

In addition, the potential of collard green leaves (CGLs) in the food industry is being examined through their application in convenience food, offering convenient consumption options that minimize the time and effort required for cleaning, preparing, and consuming plant-based foods. This attention is also motivated by the results of a recent study of 2024 that highlights the low levels of chlorophyll intake among certain age groups in the European population; in particular, Italian children have the lowest reported consumption [16]. Given the proven health benefits of chlorophyll, this research also investigates variations in chlorophyll content in different CGL preparations, including fresh-cut, fifth-range (cooked and vacuum-packed), and freeze-dried formats. The aim is to identify simple methods to increase chlorophyll intake in daily diets, thus making chlorophyll consumption more accessible and practical, while also promoting greater consumer confidence in these alternatives.

## 2. Materials and Methods

### 2.1. Plant Material

All the CGLs, cultivar “Couve-Manteiga”, were supplied by Azienda Agraria Evangelisti (Cesena, Italy), with the harvesting site located at a latitude of 44°8′0″ N and a longitude of 12°14′0″ E. The same company directly provided a sample of 5 kg of fresh-cut CGLs and provided a sample of 2 kg of the parts discarded during sorting, such as the outermost and hardest leaves, those damaged or with shape defects. A total of 2 kg of samples of the fifth range, pasteurized and vacuum-packed, was supplied by the Ghisetti company (Badia Polesine, Rovigo, Italy), specialized in the production of ready-to-eat vegetable products. All samples were transported to the laboratory in thermal boxes.

### 2.2. Preparation of Freeze-Dried CGLs

To ensure adequate representativeness, 100 leaves were randomly selected from the fresh-cut sample received and freeze-dried at a pressure of 28 mbar, at –50 °C, for four days, using a freeze-dryer (Edwards Italy, Milan, Italy). After the freeze-drying phase, the samples were ground with a mortar and pestle to obtain a homogeneous sample with a particle size of less than 500 μm. Then, the powder was vacuum-packed and stored at –20 °C until further analysis. The same process was applied to the by-products, freeze drying the entire amount of product received.

### 2.3. Determination of Water Activity

Activity water (aw) was measured using the LabMaster-aw instrument (Novasina AG, Lachen, Switzerland) with an accuracy of ±0.01 at 25 °C. After calibration, the samples were placed in a sampling chamber until the equilibrium was reached. Each measurement was performed in triplicate.

### 2.4. Proximate Composition of CGLs

Moisture was measured using the oven-drying method at 105 °C for 24 h. Ash content was measured by weighing the samples before and after burning them in a muffle furnace at 550 °C for 6 h, as described in Methods 942.05 and 934.01 by the Association of Official Analytical Chemists (AOAC). Crude protein content was evaluated using the Kjeldahl method, and the results were multiplied by the nitrogen conversion factor of 6.25 (AOAC, Method 981.10). The crude fiber was evaluated by boiling the leaves in 0.26 M sulfuric acid for 30 min. The insoluble residue obtained was filtered and washed, and the filtrate was boiled in 0.31 M sodium hydroxide and filtered and rewashed. The final filtrate was dried at 130 °C for 120 min. Weight loss was measured at 350 °C. Crude lipid content was determined according to the Soxhlet technique, using a Soxtec™ 2046 extraction system (FOSS, Hillerød, Denmark). Available carbohydrate was obtained by difference [48,49], and, finally, the energy value was calculated using Equation (1):

(1) $$\begin{array}{l} Energy\;\left(\frac{kcal}{100g\;product}\right)\\ \;\;\;\;=4\times \left(g\;protein+g\;available\;carbohydrates\right)\\ \;\;\;\;+2\times \left(g\;dietary\;fiber\right)+9\times \left(g\;fat\right) \end{array}$$

### 2.5. Amino Acids Profile Determination

Amino acids were analyzed following a method adapted from European Pharmacopoeia and previously described by Ebrahimi et al. (2022) [50]. For the separation and quantification of amino acids (AAs) in CGLs, an Agilent 1260 Infinity High-Performance Liquid Chromatography (Agilent, Santa Clara, CA, United States) with a reversed-phase C18 column (CORTECS C18, 2.7 µm, 2.1 × 150 mm), maintained at 45 °C, and a diode array detector (Agilent 1260 Series, DAD VL+) was used. AAs were analyzed after acid hydrolysis and pre-column derivatization with 6-aminoquinolyl-N-hydroxysuccinimidyl carbamate, separated by RP-HPLC, and analyzed by UV detection, following the method described by Bosch et al. (2006) [51]. Briefly, for amino acid determination, the sample protein was hydrolyzed with 6 M acid hydrochloride at 105 °C for 24 h. To determine cysteine (Cys), a method involving a reaction with 3,3-dithiodipropionic acid to form a mixed disulfide was used, followed by acid hydrolysis. After hydrolysis, the samples were neutralized with 8 M sodium hydroxide, adjusted to volume, and filtered through 0.45 µm filters. Next, the derivatization step was conducted by adding AccQ-Tag Ultra borate buffer and the filtered sample, followed by adding the derivatization agent dissolved in acetonitrile and heating for 10 min at 55 °C. The sample was then diluted and injected into HPLC. Tryptophan (Try) was determined following a method adapted from Directive 2000/45/EC. The sample was hydrolyzed in Teflon vials with barium hydroxide and water at 105 °C for 24 h and then neutralized and diluted with a 1 M sodium borate buffer. After filtration, the sample was injected into the column (Xselect HSS T3, 5 µm; 4.6 × 250 mm), and separation was performed by an isocratic elution system consisting of sodium acetate/acetonitrile.

### 2.6. Fatty Acids Profile Determination

Fatty acids (FAs) content in CGLs was analyzed by two-dimensional gas chromatography (GC × GC) using an Agilent 7890A gas chromatograph with an Agilent 7683 autosampler, an Agilent Flame Ionization Detector (FID), and an Agilent CFT modulator. To prepare the sample for FAs analysis, 40 mg of CMLs was added to 1 mL of sodium methoxide in MeOH (0.5 M), followed by incubation at 50 °C for 15 min and cooling to room temperature. Next, 1.5 mL of MeOH containing 5% HCl was added, and the mixture was incubated at 80 °C for 15 min, followed by cooling to room temperature. After that, 2 mL of hexane and 2 mL of 6% potassium carbonate were added to the cooled mixture. After vortex agitation for 30 s and centrifugation at 4000× *g* at 4 °C for 5 min, the supernatant containing fatty acid methyl esters (FAMEs) was injected into the GC. The GC temperature program was set with an initial oven temperature of 40 °C (holding time of 2 min), followed by heating to 170 °C at a rate of 50 °C/min (holding time of 25 min) and then increasing the temperature to 250 °C at a rate of 2 °C/min (holding time 14 min). The injection port and detector temperatures were 270 °C and 300 °C, respectively. The volume injected was 1 µL in split mode (split ratio 160:1), using hydrogen as the carrier gas. The columns used were a Supelco SP-2560 (Merck, Darmstadt, Germany) as the primary column and an Agilent J&W HP-5 ms as the secondary column. The resulting two-dimensional chromatograms were processed with GC × GC Image R 2.2 software from Zoex Corp., Houston, TX, USA. Individual FAs were expressed as a percentage of TFAs. Chemicals used as standards were high-purity grade and were purchased from Sigma-Aldrich (Merck, Milano, Italy).

### 2.7. Laboratory Sous Vide Cooking of CGLs

In order to monitoring the variations in color and chlorophyll content due to cooking, the fresh-cut leaves were weighed (20 g) and vacuum-sealed in ORVED vacuum-embossed kitchen bags (30 cm × 25 cm) with layers of OPA/PP and oxygen permeability of 30 cm^3^/m^2^/24 h/bar (23 °C, 50% RH), using an automatic packaging machine (Dito Sam, mod 600528, Pordenone, Italy). The vacuum-packed CGLs were placed in a water bath maintained at the selected temperature of 100 °C. The samples were heated for 5, 10, 15, 20, and 25 min, respectively. The untreated sample was the control. As soon as the treatment was completed, the samples were immersed in a basin with water and ice to stop further post-cooking biochemical changes. The vacuum packets were used for the determination of colorimetric parameters and chlorophylls of CGLs.

### 2.8. Colorimetric Properties of CGLs

Colorimetric parameters were evaluated using the Chroma Meter CR-300 colorimeter (Konica Minolta, Milan, Italy), set to illuminant D65 and an observation angle of 10°. Prior to testing, the device was calibrated with a standard white tile (L* = 84.1, a* = 0.32, b* = 0.33; Konica Minolta, Milan, Italy). The negative value of a* was considered the parameter of green (−a*). For each sample, 10 measurements were taken. For the leaves, both fresh and cooked, measurements were made with a measuring area of 8 mm in the leaf blade, while values on the freeze-dried powder were taken over a homogeneous area of 50 mm.

### 2.9. Determination of Chlorophyll

The extraction was performed according to the methods reported by Ebrahimi et al. (2024), with some modifications [52]. As an extraction solvent, 75% EtOH was used, and 2 g of ground sample was added to a falcon containing 20 mL solvent. The solid/liquid ratio was 1:10 (*w*/*v*). The spectrum of the extract was recorded from 400 to 700 nm, with reading ranges of 5 nm, using a spectrophotometer (Varian Carry 50 Bio UV/Vis, Agilent Technologies, Santa Clara, CA, USA). The absorbance values at 664 nm and 648.6 nm were recorded separately. The concentration of Chl_a_ and Chl_b_ was determined spectrophotometrically, using the method described by Lichtenthaler and Buschmann (2001) [53]. Since Chl_a_ and Chl_b_ have no absorbance at 750 nm, each absorbance value was corrected by subtracting that of the mixture at 750 nm from the measured absorbance. The contents of Chl_a_ and Chl_b_ were calculated using Equations (2) and (3), respectively. Total chlorophyll is given by the sum of chlorophyll a and b.

(2) $${Chl}_{a}\left(\frac{\mu g}{g}\right)=\frac{(13.36\cdot (A_{664.1}-A_{750})-5.19\cdot (A_{648.6}-A_{750}))\cdot V_{ex(mL)}}{w_{ex\;(g)}}$$

(3) $${Chl}_{b}\left(\frac{\mu g}{g}\right)=\frac{(27.43\cdot (A_{648.6}-A_{750})-8.12\cdot (A_{664.1}-A_{750}))\cdot V_{ex\left(mL\right)}}{w_{ex\;\left(g\right)}}$$

where Chl_a_ represents chlorophyll a; Chl_b_ is chlorophyll b; *A* is the absorbance at 664.1, 648.6, and 750 nm; *V*_ex_ is the volume of solvent in the extraction; and *W*_ex_ is the weight of the sample in the extraction.

### 2.10. Data Analysis

All analyses were performed in triplicate (*n* = 3), except for colorimetric measurements, which were taken tenfold (*n* = 10), for statistical analysis, and the results were expressed as a mean ± standard deviation. The data collected were analyzed using Excel^®^ for Microsoft 365 (Microsoft, Redmond, WA, USA) and Origin Pro 2024 (OriginLab, Northampton, MA, USA). An analysis of variance (ANOVA) was performed on the data, and Tukey’s test was used for comparisons, with significance and confidence levels set at 0.05 and 95%, respectively. The charts were generated using Origin Pro 2024 (OriginLab, Northampton, MA, USA).

## 3. Results and Discussion

### 3.1. Proximate Composition

The proximate composition of the product is shown in Table 1. All data are expressed as 100 g of fresh weight (FW), specifically referring to the edible part of the product. The CGLs have a high water content (88.94 ± 0.55 g/100 g FW) and an aw of 0.9813 ± 0.0046. The fat content is low (0.94 ± 0.5 g/100 g FW), and the FAs profile is characterized by the prevalence of polyunsaturated fatty acids (PUFAs). Dietary fibers are the most abundant component (3.39 ± 0.08 g/100 g FW), followed by proteins (3.01 ± 0.04 g/100 g FW), which contribute significantly to the caloric value of the vegetable (30.66 ± 0.21 kcal/100 g FW), which nevertheless remains a low-calorie product [54]. Available carbohydrates are very low (0.85 ± 0.05 g/100 g FW). Proteins account for most of the weight and contribute 39.15% to the product’s energy, making this vegetable a “source of protein” product. For every 100 kcal, 11.06 g of total fiber is provided, making the product also a “source of fiber” [54]. CGLs contain slightly more protein (3.01 ± 0.04 g/100 g FW) than other green leafy vegetables commonly consumed in Italy, such as cabbage (2.1 g/100 g FW), lettuce (1.8 g/100 g FW), arugula (2.6 g/100 g FW), and endive (0.9 g/100 g FW) [55].

Italian guidelines recommend consuming at least five portions of fruit and vegetables a day [32]. According to these guidelines, a serving of vegetables corresponds to about 200 g, which would translate into 6.77 g of dietary fiber provided by one serving of CGLs, equaling just over one-fourth of the official recommendations that suggest a dietary fiber intake of 25 g per day for adults [55]. In addition, CGLs also contain slightly more fiber (3.39 ± 0.08 g/100 g FW) than other varieties of the same species commonly consumed in Italy, such as cauliflower (2.4 g/100 g FW), cabbage (2.6 g/100 g FW), or broccoli (3 g/100 g FW) [55].

### 3.2. Soluble Sugars

As regards the composition of soluble sugars (Table 2), the main sugar detected was glucose (0.44 ± 0.01 g/100 g FW), followed by fructose (0.18 ± 0.03 g/100 g FW) and rhamnose (0.08 ± 0.01 g/100 g FW). Glucose is the most abundant soluble sugar, as demonstrated also by a recent study on the phytochemical composition of selected genotypes of organic kale [6]; however, this finding is in contrast with what was reported by another study, where fructose was found to be the most abundant sugar [56]. It is also important to note that both the stage of development of the plant and the environmental conditions affect both the profile and the amounts of sugars. In this respect, it has been reported that sucrose decreased during the development of the plant, whereas fructose increased in cabbages grown at 2 °C, improving their sweetness [57].

### 3.3. Mineral Composition

The mineral composition of CGLs is shown in Table 3. Calcium (333.09 ± 1.020 mg/100 g FW) was the main mineral found in the analyzed samples, followed by potassium (215.53 ± 1.06 mg/100 g FW) and sulfur (108 ± 0.19 mg/100 g FW). The latter, typical of the genus *Brassica*, is part of many bioactive components important for health, the first of all glucosinolates in Cruciferous vegetables derived from the breakdown of some isothiocyanates [58].

The calcium content in 100 g of this product is significantly higher than that found in other green leafy vegetables commonly consumed in Italy, such as endive (93 mg/100 g FW), lettuce (45 mg/100 g FW), and spinach (78 mg/100 g FW). It also surpasses the calcium levels typically found in other Brassica species, which a range from 30 to 60 mg/100 g FW [55].

A 200 g serving of CGLs would provide 666.18 mg/100 g FW of calcium and 431.05 mg/100 g FW of potassium, covering 83% of the daily requirement of calcium (800 mg/day) and just over 20% the daily requirement of potassium (2000 mg/day) [59]. One portion is therefore an important source of calcium and potassium [54].

This combination of minerals supports several vital functions, contributing to the overall well-being of humans [60]. Plant-based nutrition is becoming increasingly popular among athletes; it is critical that these diets meet the rigorous nutritional requirements needed to ensure optimal performance and effective recovery. Among the essential micronutrients that vegan athletes need to pay special attention to are iron, vitamin B_12_, calcium, vitamin D, zinc, and omega-3 fatty acids [61]. Calcium is crucial for bone health, preventing conditions such as osteoporosis, and for vital functions such as muscle contraction, nerve transmission, and blood clotting. In addition, it plays an important role in glucose regulation and thyroid function, both of which are crucial for energy metabolism and athletic performance. To ensure adequate calcium intake, athletes must include calcium-rich vegetables in their diet, such as kale, spinach, cabbage, broccoli, artichokes, and green turnips [61,62]. Considering the high calcium content in CGLs, concentrated solutions of the CGLs as freeze-dried products to be used to fortify snacks or energy drinks for athletes could be a way to ensure a constant intake of calcium even in these diets with dietary restrictions. Furthermore, it emerges that, in CGLs, the ratio of sodium to potassium is less than 0.6 mg/mg, suggesting that this vegetable is suitable for hypertensive consumers [63]. Magnesium (29.45 ± 0.07 mg/100 g FW) is a very important macronutrient for plants, as it is a structural element of the chlorophyll molecule; it activates more than 300 enzymes and contributes to the stabilization of some subcellular structures, such as ribosomes; and it also plays an important role in plant photosynthesis, carbohydrate transport, and nucleic acid and protein synthesis, as well as in the generation of reactive species oxygen [64]. Since magnesium deficiency frequently occurs in old age, a balanced magnesium intake may contribute to healthy aging [65]. A 200 g serving of CGLs has 58.90 mg/100 g FW of magnesium, 15.7% of the daily requirement [54,59].

### 3.4. Amino Acid Profile

Table 4 summarizes the content of the 18 AAs commonly found in proteins. For a better explanation, the identified amino acids were divided into two groups: essential amino acids (EAAs) and non-essential amino acids (NEAAs). The nine EAAs are leucine (Leu), isoleucine (Ile), valine (Val), phenylalanine (Phe), threonine (Thr), tryptophan (Trp), Methionine (Met), lysine (Lys), and histidine (His). Conversely, alanine (Ala), serine (Ser), proline (Pro), arginine (Arg), aspartic acid (Asp), tyrosine (Tye), glutamic acid (Glu), and cysteine (Cys) are NEAAs [66,67,68].

Histidine, although synthesized by the body, in some conditions, such as chronic obstructive pulmonary disease (COPD) and chronic kidney disease (CKD) [69,70], is not produced in sufficient quantities to meet physiological needs, making it an indispensable amino acid. Its deficiency can lead to significant reductions in hemoglobin levels [68,70]. Furthermore, supplementation with doses of 4.0–4.5 g histidine and increased dietary histidine intake are associated with decreased BMI, adiposity, markers of glucose homeostasis (e.g., HOMA–IR, fasting blood glucose, and 2 h postprandial blood glucose), proinflammatory cytokines, and oxidative stress [70]. The sum of total amino acids (TAAs) in CGLs is 2746.91 ± 18.96 mg/100 g FW, that of the NEAAs is 1598.96 ± 0.34 mg/100 g FW, and that of the EAAs is 1147.95 ± 19.30 mg/100 g FW. These data indicate a good ratio of EAAs/NEAAs in CGLs. According to the ideal amino acid composition proposed by the Food and Agriculture Organization of the United Nations (FAO)/WHO/United Nations University (UNU), the EAA/TAAs ratio should be ≈40%, and that of the EAA/NEAA should be ≥ 60% [69]. In the present study, whole leaves were examined, but previous studies have shown that leaf blades contain more amino acids than the parts of stems involved in nutrient transport [71,72].

The most abundant AAs are acidic AAs Glu and Asp, respectively, at 470.25 ± 5.20 mg/100 g FW and 324.62 ± 0.53 mg/100 g FW, as also reported in previous studies [56,72,73]. In contrast, the AAs present at the lowest quantities in CGLs are the sulfur AAs Cys (31.36 ± 0.23 mg/100 g FW) and Met (29.59 ± 5.47 mg/100 g FW), as reported in previous studies too [56,72,74,75]. Table 5 reports the EAAs content of CGLs in comparison with the recommended daily allowances (RDA, in a reference 70 kg man) of EAAs, according to the FAO/WHO/UNU (2007), for adults (>18 years) [68]. The AAs profile of CGLs in respect to RDA was also compared with the profiles of other plants, as previously reported [76,77]. Among vegetables, soy is known to have an AAs profile close to that of animal proteins, except that it has a lower content of sulfur AAs [78,79]. As a matter of fact, among vegetables, soy is the closest to the RDA, providing per 100 g of edible product an excess of all the EAAs, both as individual EAAs and as their sum. Soy is the legume with the highest amount of protein [55,80]. In contrast, for the same amount of edible product, beans (legumes) and wheat (cereals) proved to be much more deficient than soybeans, both in terms of individual amino acids and their sum. Cereals are limited in lysine content, whereas all legumes are deficient in sulfur amino acids [78,80]. Other vegetables have a much lower protein content than even cereals and legumes do, and, consequently, 100 g of their edible part is even more away from the RDA of a 70 kg man. 

However, in CGLs, the amino acids content reported as mg/g of protein and the amino acids profile indicated by the WHO/FAO/UNU (2007) [68] interestingly indicate that all the essential amino acids are provided in sufficient or even abundant amounts compared to the EAAs content of the “ideal” protein, with the exception of [Met + Cyt], which, however, is only marginally reduced (Table 6). The relative contents of His, Lys, [Phe + Tyr], Trp, and Thr are particularly abundant, and the sum of EEAs (mg/g protein) is 1.5 times higher than that shown in the WHO/FAO/UNU indices (2007) and significantly higher than that in other vegetables, such as wheat, potatoes, spinach, and cauliflower, which are limited in several essential amino acids. On the basis of these evaluations, concentrating the dry matter of CGLs via freeze drying and, thus, increasing the protein content could result in functional ingredients with an extremely favorable amino acids profile.

These considerations have been made on the fresh product, but although it can be eaten raw, thinly sliced, or in centrifuges [81], generally, consumers tend to consume this type of vegetable cooked. Cooking can cause changes in the nutritional value of protein, decreasing the AAs content. A previous study conducted by Lisiewska et al. (2007) [72] showed that the cultivar *Winterbor* F1 kale boiled for 15 min had about 78% of the TAAs content found in fresh leaves, where the most important decreases were in the AAs Val, Ile, Cys, Met, Ala, and Phe. The authors report that the loss of TAAs was broadly similar to the loss of dry weight (DW) mainly influenced by the leaching of constituents during cooking. In this sense, vacuum packs in sous vide cooking reduce the release of components such as AAs or organic acids [82].

### 3.5. Fatty Acid Composition and Content

As shown in Table 7, in CGLs, the predominant FAs are unsaturated fatty acids (USFAs), categorized into 58.87% PUFAs and 22.80% monounsaturated fatty acids (MUFAs). Meanwhile, saturated fatty acids (SFAs) account for only 18.34% of the total fatty acids (TFAs), with palmitic acid (C16:0) being present in the highest amount. The balance is therefore totally shifted toward USFAs, with PUFAs prevailing where α-linolenic acid (C18:3n3) of the omega-3 series (ω-3) and linoleic acid (C18:2n6) of the omega-6 series (ω-6) are the main representatives, making up, respectively, 42.28% and 12.07% of the total fatty acids. α-Linolenic acid (C18:3n3) is the fatty acid present in the highest percentage of the TFAs. These results are consistent with those identified in a previous study of *Brassica oleracea* var. *acephala* [56]. It is widely recognized how crucial a healthy diet is to human health. Unbalanced diets are associated with an increase in lifestyle-related diseases, which are particularly common among populations in industrialized countries [83,84,85,86,87]. The importance of proper dietary FAs intake is well documented and relevant to overall health. Diets in industrialized countries tend to include excessive amounts of SFAs at the expense of USFAs. This imbalance contributes to the increase in chronic non-communicable diseases related to lifestyle [83,88,89,90]. The WHO recommends that adults reduce their intake of SFAs to 10% of total energy. In addition, the WHO dietary fat guidelines suggest a further reduction in SFAs intake to below 10% and replacing them with PUFAs and MUFAs from plant sources or fiber-rich carbohydrates [91]. Essential fatty acids (EFAs) refer to those PUFAs that must be provided by foods because they cannot be synthesized in the body but are necessary for health. There are two families of EFAs, ω-3 and ω-6, based on the position of the first double bond from the ω end of the fatty acid [92]. In both families, there are many forms of PUFAs: α-linolenic acid (ALA), eicosapentaenoic acid (EPA), and docosahexaenoic acid (DHA) of the ω-3 family; and linoleic acid (LA), dihomo-γ-linolenic acid (DGLA), and arachidonic acid (AA) of the ω-6 family are the PUFAs that are important for human health. Both ω-3 and ω-6 are competitively metabolized by the same set of enzymes. Excessive consumption of ω-6 with low ω-3 intake is strongly associated with the pathogenesis of many chronic diseases related to the modern diet [93]. Currently, the ratio of the PUFAs ω-6 to ω-3 is between 20:1 and 50:1, much higher than the recommended ratio of 4:1 or 5:1 [32]. Due to this imbalance, higher amounts of lipid mediators derived from LA and AA are produced, which are responsible for the formation of thrombi and atheromas, allergic and inflammatory disorders, cell proliferation, and overactivity of the endocannabinoid system [85,89,92]. Although the FAs levels in CGLs are not high since they contain an average of 1% fat on fresh product, it is interesting to note that the FAs profile is advantageous with a ω-3/ω-6 ratio of 3.2.

### 3.6. Chlorophyll Content in CGL Convenience Food

**Figure 1:**
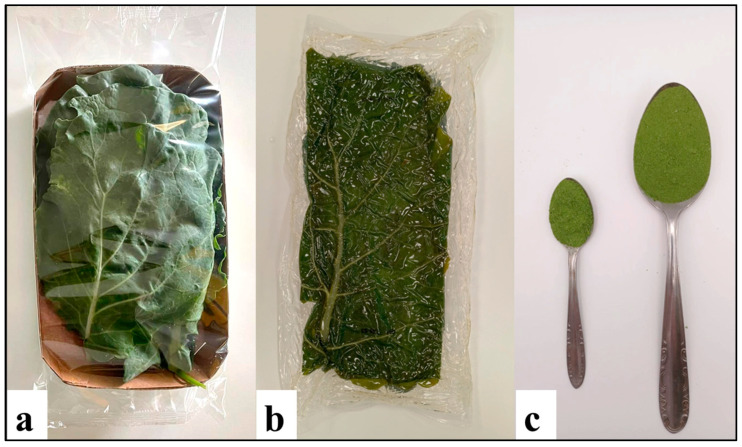
Convenience foods obtained from CGLs: (**a**) fresh-cut product selected, sorted, cleaned, washed, dried, and packaged (200 g packages); (**b**) fifth-range product vacuum-cooked in an industrial autoclave (200 g packages); and (**c**) freeze-dried product, one teaspoon (5 g) and one tablespoon (15 g). (Photos taken by the authors.)

In light of the growing evidence of the health benefits of chlorophyll pigments and the limited consumption of these important pigments in many European countries, especially among children [16], the study aimed to explore the chlorophyll content in CGLs in practical and ready-to-use solutions for the modern consumer, who has little time to devote to food preparation and the search for healthy, fast, and convenient solutions [45,46,94]. In this regard, fresh-cut [95], fifth-range, and freeze-dried CGLs were considered (Figure 1).

In the present study, the CGLs analyzed were vacuum-cooked in an industrial autoclave, with an equivalent heat treatment ≥10 min at 90 °C at the core of the product, capable of bringing at least 6 decimal reductions in the spores of non-proteolytic *Clostridium botulinum* microorganisms, taken as a reference for Refrigerated Processed Foods of Extended Durability (REPFEDs) [43,96,97]. Fresh plant-based foods are not always available all year round, and due to their high water content and aw value, they are very perishable. In this respect, the aw of fresh CGLs was 0.98 ± 0.01. For this reason, drying can allow for long-term consumption and facilitates handling, transport, and storage, and freeze drying is especially known for producing high-quality food powders [23]. The lyophilized CGL powders showed an aw of 0.31 ± 0.01. Since at an aw value of 0.61, there is no bacterial proliferation, products below this activity water range have a long shelf-life [98,99]. Moreover, freeze-dried powders based on vegetables and fruits can be used to obtain instant drinks with high nutritional value [100], additions to soups, confectionery, breakfast cereals, baked goods, snacks, condiment sauces, etc. [23,100]. In addition, freeze drying makes it possible to obtain value-added products even starting from plant by-products [101,102,103,104,105]. In fact, cutting residues and the hardest and most damaged outer leaves were used to obtain the freeze-dried CGLs. Table 8 shows the Chl_a_, Chl_b_, and total Chl (Chl_a+b_) content, expressed in μg/g FW; and the greenness parameter (−a* value) of the products concerned. For the fifth-range and the freeze-dried products, the % of chlorophyll retention compared to the fresh-cut product on a DW basis is also reported.

Photosynthetic pigments, such as chlorophylls and carotenoids, are essential for photosynthesis and contribute significantly to plant color. Chlorophylls, mainly Chl_a_ and Chl_b_, absorb blue and red light, giving the leaves their characteristic green color. In addition to capturing light for photosynthesis, these pigments protect plants from oxidative damage due to their antioxidant properties [15,17,106]. The sous vide-pasteurized product in the industrial autoclave had a total chlorophyll retention of about 83.5% compared to the fresh product. The freeze-dried product had more than ten times the total chlorophyll content compared to the fresh product, and when comparing the values based on the dry weight, freeze drying resulted in an overall chlorophyll retention of 97.66%. Similar retention rates have been identified for freeze-dried chives [24]. As far as the green color is concerned, clearly, the cooking has led to a significant decrease (*p <* 0.05) in the greenness parameter (–a*) due to the degradation of chlorophylls and the formation of pheophytin, a phenomenon that increases with the increase in the cooking time [107]. In contrast, in freeze-dried CGLs, there is an increase in the greenness parameter (–a*) due to the drastic reduction in the volume of plant matter, which concentrates chlorophyll and other pigments, making the green color more saturated than in the fresh leaf. One teaspoon (~5 g) of freeze-dried CGLs provides 17.30 mg of chlorophyll, which is 7.30 times the current average daily consumption of Italian children and about half that of European children. One tablespoon (~15 g) provides about 51.91 mg of chlorophyll, more than 30% of the current daily consumption of adolescents, adults, and the elderly in Italy; and 34% of that of European adolescents, 25% of that of European adults, and 28% of that of the elderly in European countries [16]. The use of these chlorophyll concentrates and other bioactive compounds (polyphenols, glucosinolates, fibers, vitamins, minerals, etc.) for the enrichment of food and beverages represents an innovative and sustainable approach. This would not only allow the full use of plant by-products from the agrifood industry to produce functional ingredients but would also facilitate an increase in chlorophyll intake in the population. Such supplementation could have beneficial effects both in nutritional and public health terms, contributing to the prevention of diseases related to oxidative stress and to the improvement of the nutritional profile of foods intended for consumption. A 200 g serving of sous vide-cooked CGLs provides 71.47 mg of chlorophyll, equivalent to 30 times the current daily consumption of Italian children and 45% of that of adolescents, adults, and the elderly. At the European level, it represents two times the current daily consumption of children, 47% of that of adolescents, 34.5% for adults and 38.4% for the elderly [16]. These data raise intriguing questions about the potential impact of sous vide cooking on chlorophyll content.

The elderly represent the fastest growing segment of the population and are at the greatest risk of chronic diseases. According to the WHO, between 2015 and 2050, the percentage of the world’s population over 60 will almost double, from 12% to 22% [108]. Although the consumption of fruits and vegetables is protective, many people do not reach the recommended levels and prefer starchy vegetables to more nutritious ones, such as dark green and orange vegetables. Older age is often associated with decreased appetite and oral health problems, which can further limit the intake of fruits and vegetables. Additionally, older adults with disabilities or functional limitations face specific difficulties in buying, preparing, and consuming fresh food. Deterioration of physical health, as well as loneliness, in the elderly is generally accompanied by a decline in diet quality, further aggravating nutritional risks. Finally, many older adults who live alone and who consume small portions tend to avoid large packages to prevent waste, especially in regard to vegetables [109,110,111,112]. The introduction of single-serving solutions of green leafy vegetables, already cooked and, therefore, with a softer consistency would represent an advantageous nutritional option for the elderly. These products, easily stored in the refrigerator and characterized by a long shelf-life, i.e., of several weeks or months [113,114,115], would reduce the need for frequent purchases and simplify vegetable intake for individuals with mobility limitations or difficulties in food preparation. In addition, these products, rich in essential nutrients, such as chlorophyll, vitamins, and minerals, could help improve the intake of micronutrients, facilitating the increase in vegetable consumption among the elderly population. A 200 g serving of fresh-cuts CGLs would provide 36 times the amount of chlorophyll currently consumed daily by children in Italy and almost double the current average daily consumption of children in Europe. In addition, a single serving would account for between 53% and 56% of the current daily consumption of chlorophyll for adolescents, adults, and the elderly in Italy, and between 41% and 56% for the same age groups in Europe [16]. However, these last considerations refer to the consumption of fresh leaves, for example, in salads or smoothies. Cooking leads to a reduction in chlorophyll content and, consequently, the green pigmentation to varying degrees, depending on several factors. These include the cooking method applied, the cooking duration, the cooking temperature, the pH of the cooking medium, the presence of salts or other additives, etc. [106,116,117,118].

### 3.7. Impact of Sous Vide-Cooking Processing on Color and Chlorophyll Content

Many vegetables are consumed after different cooking methods that induce chemical and physical changes to the product, affecting the bioavailability and the content of chemopreventive compounds in vegetables [106,119,120]. Chlorophyll is the pigment responsible for the green color of plants and is a very important factor that influences the quality of vegetables. Generally, plants have Chl_a_ and Chl_b_ in a ratio of 3:1, which can vary due to environmental factors, growing conditions, and high sun exposure [15,121,122]. Weak light transmission (shading) typically results in a decrease in the proportion of red light absorbed by Chl_a_ and an increase in the proportion of blue light absorbed by Chl_b_ [123]. Chl_b_ is more stable than Chl_a_ during thermal processing, and Chl_a_ is more susceptible to pheophytinization during heating and is even more susceptible under acidic conditions [116,117,124,125,126]. When the temperature exceeds 60 °C, the membranes surrounding the chloroplast begin to become damaged, exposing the chlorophyll to the plant’s natural acids [127]. In the sous vide treatment, many soluble nutrients are retained that would be leached by cooking in water; however, the latter would also allow the dispersion in water of organic acids that are retained in the sous vide treatment and influence the degradation of chlorophyll through a slight lowering of the pH [53,116]. Table 9 reports the changes in Chl_a_, Chl_b_, and total chlorophyll (Chl_a+b_) content, expressed in μg/g FW; ratio of Chl_a_ to Chl_b_ (Chl_a_/Chl_b_); greenness parameter (−a* value); and pH for samples of CGLs (20 g) cooked sous vide in boiling water (100 °C) for 5, 10, 15, 20, and 25 min, respectively.

**Figure 2:**
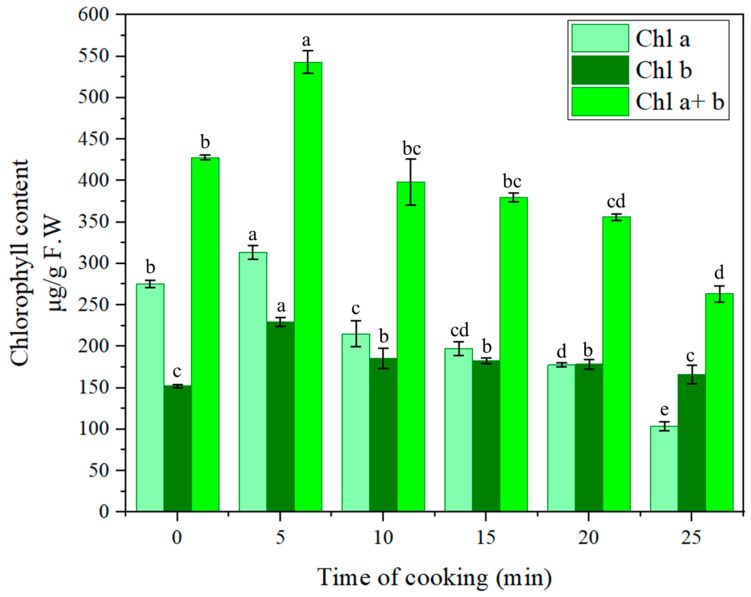
Change in chlorophyll content (Chl_a_, Chl_b_, and Chl_a+b_) as a function of sous vide cooking time (5, 10, 15, 20, and 25 min), expressed in μg/g FW. Different letters indicate statistically significant differences between samples at different cooking times (*p <* 0.05), according to the ANOVA test, followed by Tukey’s test.

As the data reported in Table 8 show, changes in chlorophyll content are reflected in changes in green color parameters. There is an initial increase in green color and chlorophyll content in the first 5 min of treatment and then a gradual decrease that has a more important impact on Chl_a_ than Chl_b_, progressively lowering the Chl_a_/Chl_b_ ratio (Figure 2).

**Figure 3:**
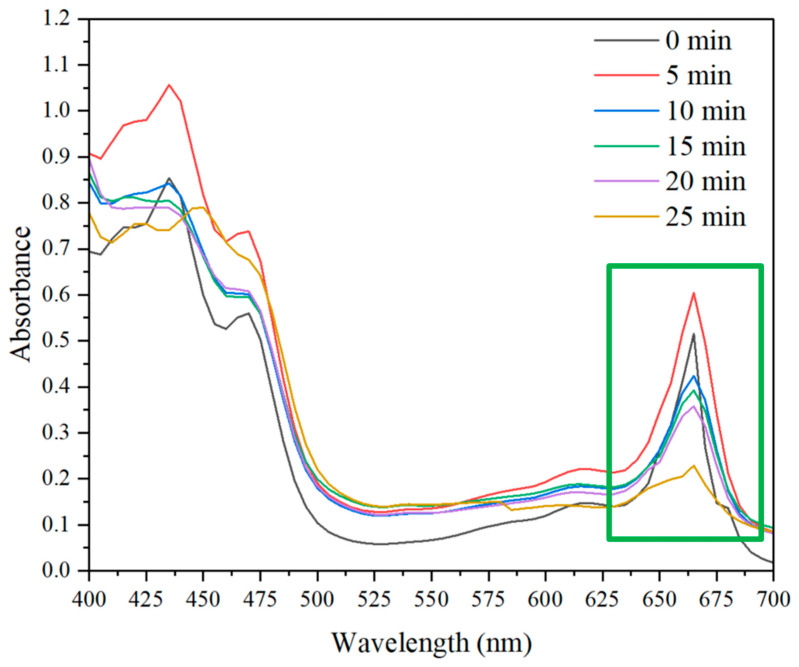
The chlorophyll absorption spectrum was measured at different sous vide cooking times (5, 10, 15, 20, and 25 min) in the wavelength range between 400 and 700 nm. The control (0 min) is represented by fresh-cut CGLs that are not cooked. The absorbance in the red region (650–680 nm) is highlighted in the green box.

As widely reported by previous studies, in the first minute of heat treatment, there is the removal of air around the fine hairs on the surface of the plant and the expulsion of air between the cells. This would lead to an alteration of the reflective properties of the surface, resulting in a brighter green [128,129,130,131]. Furthermore, this increase is also due to the initial effect of cooking that softens plant tissues, facilitating the extraction of bioactive compounds, such as chlorophylls, from the cellular matrix [132,133,134]. After the first 5 min, the bright green color begins to degrade and turns more and more toward olive green. The higher the temperature, the greater the increase in color, but at the same time, the sooner the color begins to decay [128]. Chemical reactions are triggered that lead to the formation of chlorophyll derivatives, mainly chlorophyll isomers (chlorophylls a’ and b’) and pheophytins a and b, phenomena also encouraged by the slight and gradual lowering of pH, also found in previous studies [40]. Chlorophylls a’ and b’ have the same absorption spectra, and therefore their formation does not cause color changes. However, the formation of pheophytins, caused by the exchange of Mg^2+^ with H^+^ at the center of the porphyrin ring of chlorophyll, is accompanied by a change in color from bright green to olive brown [128,135] with a relative drop in absorbance in the red region (650–680 nm) [53,136] of the absorption spectrum, as shown in Figure 3.

## 4. Conclusions

This study highlights the nutritional value of the leaves of the collard green (*Brassica oleracea* var. *viridis*) cultivar “Couve-Manteiga” and their potential as convenience foods to increase the intake of essential nutrients, especially chlorophyll, in the daily diet. CGLs’ profile of being low in calories and high in fiber, protein, and minerals such as calcium and potassium, support its suitability as a functional food. Chlorophyll, an essential bioactive compound found in satisfactory amounts in CGLs, is essential for reducing oxidative stress, preventing DNA damage, and offering potential antitumor benefits. The high chlorophyll retention rates achieved through sous vide cooking and freeze drying demonstrate the effectiveness of these methods in preserving the nutritional value of chlorophyll, making them suitable for the development of practical and nutrient-rich food products. A single serving of sous vide CGLs provides ≈ 45% of the current daily consumption of chlorophyll in adolescents, adults, and the elderly in Italy, while freeze-dried products can provide even higher concentrations. A teaspoon provides 7.30 times the average current daily consumption of Italian children, while a tablespoon provides just over 30% of the daily consumption of Italian adolescents, adults, and the elderly. These findings make collard green-based convenience foods ideal for filling dietary gaps in chlorophyll consumption, particularly in children and older populations. This study suggests that incorporating chlorophyll-rich convenience foods into daily meals could offer significant health benefits, promoting better oxidative defense mechanisms and overall nutritional status in different population groups.

A potential limitation of this study is its focus on the specific cultivar “Couve-Manteiga” cultivated in Italy. While the results offer valuable insights into this cultivar’s nutritional profile and chlorophyll retention, their generalizability to other collard green varieties or different geographic regions may need to be improved. Variability in climate, agricultural practices, and genetic differences among cultivars could influence the nutritional profile and bioactive compound stability. Future research is needed to explore these variations and determine whether similar conclusions can be extended to other varieties and regions. Additionally, future studies should assess chlorophyll’s stability during the shelf-life of these convenience foods. Monitoring how storage conditions, packaging, and environmental factors affect chlorophyll degradation will provide valuable insights for optimizing these products for maximum health benefits. Exploring the long-term health impact of consuming collard green-based convenience foods will further clarify their potential benefits and implications.

Finally, freeze-dried CGL powder offers promising applications in various food products, including doughs, ice creams, sauces, emulsions, and beverages. This broader utilization could enhance the nutritional profile of diverse food products, expanding the role of collard greens in improving dietary health.

## Figures and Tables

**Table 1:** Proximate composition (g/100 g FW) in CGLs.

Proximate Composition	(g/100 g FW)
Moisture	88.94 ± 0.55
Proteins	3.01 ± 0.04
Lipid	0.94 ± 0.5
Available carbohydrate	0.85 ± 0.05
Dietary fiber	3.39 ± 0.08
Ash	1.94 ± 0.03
Energy (kcal/100 g)	30.66 ± 0.21

Values are mean ± standard deviation, *n* = 3.

**Table 2:** Soluble sugars (g/100 g FW) in CGLs.

Soluble Sugars	(g/100 g FW)
Glucose	0.44 ± 0.01
Fructose	0.18 ± 0.03
Rhamnose	0.08 ± 0.01

Values are mean ± standard deviation, *n* = 3.

**Table 3:** Mineral composition (mg/100 g FW) of CGLs.

Mineral Composition (mg/100 g FW)					
Ca	333.09 ± 1.02	Si	1.53 ± 0.05	B	0.20 ± 0.01
K	215.53 ± 1.06	Fe	0.73 ± 0.02	Ba	0.08 ± 0.01
S	108.00 ± 0.19	Sr	0.62 ± 0.01	Cu	0.04 ± 0.01
Na	40.79 ± 0.01	Zn	0.32 ± 0.01		
P	38.89 ± 0.02	Mn	0.30 ± 0.01		
Mg	29.45 ± 0.07	Al	0.27 ± 0.01		

Values are mean ± standard deviation, *n* = 3.

**Table 4:** Composition of AAs in CGLs (mg/100 g FW).

AAs Profile	(mg/100 g FW)
Glutamic acid	470.25 ± 5.20
Aspartic acid	324.62 ± 0.53
Lysine *	263.31 ± 3.44
Leucine *	209.04 ± 1.16
Arginine	185.15 ± 1.69
Alanine	152.73 ± 0.62
Valine *	138.29 ± 0.85
Threonine *	133.82 ± 6.22
Glycine	132.08 ± 0.56
Phenylalanine *	129.90 ± 0.60
Serine	129.36 ± 0.34
Proline	125.32 ± 1.90
Histidine *	103.94 ± 2.49
Isoleucine *	96.43 ± 0.76
Tyrosine	48.09 ± 0.57
Tryptophan *	43.64 ± 0.16
Cysteine	31.36 ± 0.23
Methionine *	29.59 ± 5.47
Total AAs	2746.91 ± 18.96
Total NEAAs	1598.96 ± 0.34
Total EAAs	1147.95 ± 19.30
EAAs/TAAs	0.42
EAAs/NEAAs	0.72

Values are mean ± standard deviation, *n* = 3. * EAAs, essential amino acids; NEAAs, non-essential amino acids; TAAs, total amino acids.

**Table 5:** EAAs composition of CGLs compared with other plant products and with the RDA.

	RDA *	CGLs 100 g **	Soybeans 100 g	Beans 100 g	Wheat 100 g	Potato 100 g	Spinach 100 g	Cauliflower 100 g
**Protein content (g)**		3.01	36.9	10.2	13	2.1	3.4	3.2
***Amino acid* (mg)**								
**Histidine**	700	103.94	1170	303	228	28	83	37
**Isoleucine**	1400	96.43	2222	556	403	68	102	73
**Leucine**	2730	209.04	3689	885	741	96	196	126
**Lysine**	2100	263.31	3047	714	239	92	154	120
**Methionine + cysteine**	1050	60.95	1183	238	454	51	69	63
**Phenylalanine + tyrosine**	1750	177.99	3970	963	855	132	294	129
**Threonine**	1050	133.82	1843	428	310	59	113	74
**Tryptophan ^1^**	280	43.64	618	113	116	/	/	/
**Valine**	1820	138.29	2176	616	452	99	139	104
**Total EAAs**	12,880	1227.40	19,918	4816	3798	625	1150	726

* RDA for a 70 kg adult man according to the FAO/WHO/UNU (2007) [68]. ** Data are reported per 100 g of edible parts of food. ^1^ Tryptophan concentrations in potatoes, cauliflower, and spinach are not reported in References [76,77]. The protein contents of the foods are taken from published tables of CREA (Council for Agricultural Research and Analysis of Agricultural Economics) [55].

**Table 6:** EEAs profile of CGLs and other selected vegetables compared with the WHO/FAO/UNU (2007) requirements (mg/g protein).

Amino Acid Composition (mg/g Protein)								
Amino Acid	WHO/FAO/UNU Requirement *	CGLs	Soybeans	Beans	Wheat	Potato	Spinach	Cauliflower
Histidine	15.00	34.53	31.71	29.71	17.54	13.33	24.41	11.56
Isoleucine	30.00	32.04	60.22	54.51	31.00	32.38	30.00	22.81
Leucine	59.00	69.45	99.97	86.76	57.00	45.71	57.65	39.38
Lysine	45.00	87.48	82.57	70.00	18.38	43.81	45.29	37.50
Methionine + Cysteine	22.00	20.25	32.06	23.33	34.92	24.29	20.29	19.69
Phenylalanine + Tyrosine	38.00	59.13	107.59	94.41	65.77	62.86	86.47	40.31
Threonine	23.00	44.46	49.95	41.96	23.85	28.10	33.24	23.13
Tryptophan ^1^	6.00	14.50	16.75	11.08	8.92	/	/	/
Valine	39.00	45.94	58.97	60.39	34.77	47.14	40.88	32.50
Total EEAs	277.00	407.77	19,918.00	472.16	292.15	297.62	338.24	226.88

* Reference amino acid requirements for a 70 kg adult man according to of adults (FAO/WHO/UNU, 2007) [68]. ^1^ Tryptophan concentrations in potatoes, cauliflower, and spinach are not reported in References [76,77]. The protein content of the foods is taken from published tables of CREA (Council for Agricultural Research and Analysis of Agricultural Economics) [55].

**Table 7:** Fatty acid profile (% of the total fatty acid) of CGLs.

Fatty Acid	(% of the Total Fatty Acid)
C4:0: butyric acid	0.02 ± 0.01
C6:0: caproic acid	0.14 ± 0.08
C8:0: caprylic acid	0.23 ± 0.01
C10:0: capric acid	0.19 ± 0.01
C11:0: undecanoic acid	0.03 ± 0.01
C12:0: lauric acid	0.11 ± 0.01
C13:0: tridecanoic acid	0.76 ± 0.02
C14:0: myristic acid	0.32 ± 0.04
C15:0 anteiso: anteiso-pentadecanoic acid	1.66 ± 0.05
C15:0: pentadecanoic acid	0.12 ± 0.01
C16:0 iso: iso-palmitic acid	0.15 ± 0.03
C16:0: palmitic acid	11.23 ± 0.57
C17:0 iso: iso-margaric acid	0.17 ± 0.02
C17:0: margaric acid	0.20 ± 0.01
C18:0: stearic acid	2.14 ± 0.45
C20:0: arachidic acid	0.19 ± 0.01
C22:0: behenic acid	0.18 ± 0.06
C24:0: lignoceric acid	0.51 ± 0.15
C16:1 n9: palmitoleic acid	4.76 ± 0.14
C17:1 n7: heptadecenoic acid	12.52 ± 0.33
C18:1 n9: oleic acid	3.89 ± 0.86
C18:1 n7: vaccenic acid	0.91 ± 0.08
C18:1 n6: petroselinic acid	0.04 ± 0.01
C19:1: nonadecenoic acid	0.11 ± 0.02
C20:1 n9: eicosenoic acid	0.07 ± 0.02
C22:1 n9: erucic acid	0.04 ± 0.02
C24:1 n9: nervonic acid	0.47 ± 0.08
C18:3 n3: alpha-linolenic acid	42.28 ± 1.82
C20:3 n3: eicosatrienoic acid	0.21 ± 0.01
C20:5 n3 EPA: eicosapentaenoic acid	0.27 ± 0.05
C22:5 n3 DPA: docosapentaenoic acid (n–3)	0.34 ± 0.01
C22:6 n3 DHA: docosahexaenoic acid	1.89 ± 0.10
C18:2 n6: linoleic acid	12.07 ± 0.21
C18:3 n6: gamma-linolenic acid	0.11 ± 0.01
C20:2 n6: eicosadienoic acid	0.34 ± 0.05
C20:3 n6: dihomo-gamma-linolenic acid	0.68 ± 0.01
C20:4 n6: arachidonic acid	0.44 ± 0.12
C22:2 n6: docosadienoic acid	0.13 ± 0.02
C22:5 n6 DPA: docosapentaenoic acid (n–6)	±0.02
TSFA ^a^	18.34
MUFAs ^b^	22.80
PUFAs ^c^	58.87
TUSFAs ^d^	81.66
TUSFA/TSFA ^e^	4.45

Values are mean ± standard deviation, *n* = 3 on DW basis. ^a^ Total saturated fatty acid; ^b^ monounsaturated fatty acids; ^c^ polyunsaturated fatty acids; ^d^ total unsaturated fatty acids (MUFA + PUFA); ^e^ ratio of total unsaturated fatty acids to total saturated fatty acids.

**Table 8:** Chl_a_, Chl_b_, and total Chl content, expressed in μg/g FW; and greenness parameter (−a* value) of fresh-cut, fifth-range (sous vide), and freeze-dried CGLs. Also, % retention of chlorophyll compared to fresh-cut product on a DW basis.

	Fresh-cut CGLs	Fifth-Range CGLs	% Retention	Freeze-Dried CGLs	% Retention
Chl_a_ (μg/g)	275.64 ± 4.54 ^b^	218.69 ± 4.47 ^b^	83.50	2220.28 ± 46.56 ^a^	97.66
Chl_b_ (μg/g)	152.30 ± 1.90 ^b^	138.64 ± 7.86 ^b^	79.34	1240.44 ± 63.63 ^a^	97.28
Total Chl (μg/g)	427.94 ± 2.64 ^b^	357.34 ± 12.33 ^c^	91.03	3460.72 ± 23.02 ^a^	98.36
−a* value	–11.83 ± 0.19 ^b^	–9.39 ± 0.23 ^c^		–14.91 ± 0.03 ^a^	

Values are mean ± standard deviation. Means (*n* = 3) were compared by one-way ANOVA, followed by Tukey’s test (*p* ≤ 0.05). Letters indicate significant differences within a group (in alphabetical order, from the highest to lowest).

**Table 9:** Impact of the sous vide-cooking process on the content of Chl_a_, Chl_b_, and total Chl content expressed in μg/g FW; ratio Chl_a_/Chl_b_ and greenness parameter (−a* value) at different selected times. The control (0 min) is represented by fresh-cut CGLs not cooked.

	0 min	5 min	10 min	15 min	20 min	25 min
Chl_a_	275.64 ± 4.54 ^b^	313.69 ± 8.19 ^a^	215.20 ± 15.72 ^c^	197.38 ± 8.00 ^cd^	177.68 ± 2.37 ^d^	103.70 ± 5.46 ^e^
Chl_b_	152.30 ± 1.90 ^c^	229.32 ± 5.32 ^a^	185.59 ± 11.90 ^b^	182.67 ± 3.59 ^b^	178.46 ± 5.62 ^b^	166.37 ± 11.16 ^c^
Chl_a_/Chl_b_	1.81	1.37	1.16	1.08	1	0.62
Total Chl [Chl (a + b)]	427.94 ± 2.64 ^b^	543.00 ± 13.51 ^a^	398.47 ± 27.62 ^bc^	380.05 ± 5.67 ^bc^	356.14 ± 3.65 ^cd^	270.08 ± 9.68 ^d^
−a* value	–11.83 ± 0.19 ^a^	–12.23 ± 0.53 ^a^	–10.12 ± 0.46 ^b^	–8.87 ± 0.10 ^c^	–8.08 ± 0.32 ^c^	–7.93 ± 0.26 ^c^
pH	5.972 ± 0.004 ^a^	5.853 ± 0.009 ^ab^	5.823 ± 0.015 ^ab^	5.737 ± 0.017 ^b^	5.630 ± 0.018 ^bc^	5.574 ± 0.013 ^c^

Values are mean ± standard deviation. Means (*n* = 3) were compared by one-way ANOVA, followed by Tukey’s test (*p* ≤ 0.05). Letters indicate significant differences within a group (in alphabetical order from the highest to lowest).

## Data Availability

The authors declare that the data supporting the findings of this study are available within the paper. Should any raw data files be needed in another format, they will be available from the corresponding author upon reasonable request. The data are not publicly available due to privacy.
